# Renal Cell Carcinoma: Characteristics of Patients, Treatment, and Disease Progression Based on Real-World Data From a Cancer Center in Medellín, Colombia

**DOI:** 10.7759/cureus.102306

**Published:** 2026-01-26

**Authors:** Mauricio Lema, Camila Lema, Diego Morán, Mateo Pineda, Beatriz Preciado, Emilio Pérez

**Affiliations:** 1 Oncology, Clínica de Oncología Astorga, Medellín, COL; 2 Research, Clínica de Oncología Astorga, Medellín, COL

**Keywords:** disease-free survival, immunotherapy, kidney neoplasms, neoplasm staging, progression-free survival

## Abstract

Introduction

Data on demography, stage distribution, treatment, and survival rates of renal cell carcinoma (RCC) in Colombia is not known. This study aims to describe a cohort of patients with RCC treated at the Clínica de Oncología Astorga, in Medellín, Colombia.

Methods

This retrospective study included all consecutive RCC patients treated at the institution between 2008 and 2023. Clinical, treatment, and survival data were collected from medical records. For metastatic and recurrent patients, risk was dichotomized as low-risk or non-low risk. The latter included those classified as intermediate/high risk in medical records, or cases with de novo metastatic disease or progression within 12 months of initial diagnosis. Event-free survival (EFS), progression-free survival (PFS), and overall survival (OS) were estimated using the Kaplan-Meier method and subgroup analysis was performed using metastatic status. Treatment patterns were compared with the log-rank test.

Results

Data of 208 patients were analyzed. The median age was 60.4 years (interquartile range (IQR) 51.9-69.8) and 114 subjects (54.8%) were men. Clear-cell histology was available for 167 patients (80.3%). Stages I, II, III, and IV disease was observed in 58 (27.9%), 34 (16.3%), 38 (18.3%), and 48 (23.1%) cases, respectively. Metastatic disease occurred in 107 (51.4%) patients (46 synchronous and 61 metachronous). Low-risk and non-low-risk metastatic disease was detected in 43 (40.2%) and 64 (59.8%) patients, respectively. Among non-metastatic patients, 126 (95.4%) patients underwent nephrectomy. First-line treatment included tyrosine kinase inhibitors (TKI) in 58 (70.7%), immunotherapy (IO) in 12 (14.6%), and other systemic therapies in 12 (14.6%) patients; 34 (31.8%) patients received a second-line regimen, most commonly from TKI to IO. Median OS for the entire cohort was 161.1 months. OS was significantly longer in non-metastatic versus metastatic patients at diagnosis (217.0 vs. 24.9 months; p<0.001). In metastatic disease, median OS differed by risk category (low-risk, 58.4 months vs. non-low-risk, 26.6 months; p=0.01) and by first-line treatment (TKI 42.2 months, IO 18.6 months, surgery 67.3 months; p=0.03).

Conclusion

In this institution-based real-world of RCC patients from Colombia, more than half presented with or subsequently developed metastatic disease; however, only one-third were able to access second-line therapy, highlighting substantial attrition in treatment sequencing. Non-metastatic status, low-risk metastatic classification, and first-line treatment with TKI were associated with superior survival outcomes.

## Introduction

According to GLOBOCAN 2022, renal cell carcinoma (RCC) ranks as the 13th most common cancer worldwide [1]. In Colombia, RCC incidence was estimated as 2.0% and 1.5% of cancer-related deaths, corresponding to approximately 2,374 new cases and 829 deaths annually [1].

The risk factors for sporadic RCC are nonspecific and include smoking, obesity, high blood pressure, alcohol consumption, and occupational exposure to carcinogens. Hereditary RCC syndromes have also been described, accounting for a small fraction of cases. Due to its low relative frequency and the nonspecific nature of its risk factors, there is currently no established strategy for the early detection of RCC. This explains why approximately one in three cases of RCC is diagnosed at a locally advanced or metastatic stage [2]. The median age at diagnosis of RCC is 64 years. The five-year survival rates for RCC detected at localized, regional extension, and metastatic disease are 92.7%, 71.0%, and 13.9%, respectively [2].

This study aims to describe the demographic and clinical characteristics, as well as survival outcomes of 208 consecutive patients with RCC treated at Clínica de Oncología Astorga, an outpatient cancer care center in Medellín, Colombia.

## Materials and methods

This is an observational, longitudinal, retrospective study of patients with RCC in a real-world setting at the Clínica de Oncología Astorga, in Medellín, Colombia. All RCC patients treated between January 1, 2008 and January 30, 2023 at the institution were considered for inclusion.

The medical records were reviewed to identify the eligible cases. Patients aged ≥18 years were included. Patients were excluded if they did not have a histological diagnosis, if clinical data related to diagnosis or treatment were insufficient, or if they had a confirmed diagnosis of urothelial carcinoma or metastatic involvement of the kidneys originating from other primary tumors.

Data was extracted from medical records. Demographic variables were collected. Clinical data included histological type (e.g., clear-cell, papillary, sarcomatoid, etc.) and stage at initial diagnosis. Information on treatment modalities, such as surgery, systemic therapies and/or supportive care, was also gathered.

Risk classification in metastatic disease was inconsistently reported. Therefore, patients were dichotomized into low-risk and non-low-risk categories. When risk classification was unavailable, patients were assigned to the non-low-risk category if they presented with de novo metastatic disease or if progression occurred within 12 months of the initial RCC diagnosis. The non-low-risk group included patients classified as intermediate or high risk in the medical records. The remaining patients were classified as low risk.

Survival outcomes assessed included the following: Event-free survival (EFS), defined as the time from initiation of treatment with curative intent to either disease progression or death, whichever occurred first. Progression-free survival (PFS), defined as the time from the start of treatment for metastatic disease to either disease progression or death, whichever occurred first. Overall survival (OS) is defined as the time from diagnosis to death from any cause. May 30, 2025, was defined as the cut-off date for collecting information on survival outcomes. Patients whose vital status could not be confirmed were censored at the date of their last clinical contact.

The date of death was obtained from reliable sources, including medical records, ADRES (Administradora de los Recursos del Sistema General de Seguridad Social en Salud) database, or Registraduría Nacional del Estado Civil database (another governmental institution).

Data from qualitative variables were summarized using absolute and relative frequencies. Quantitative variables were expressed as median and interquartile range (IQR), depending on their distribution. Survival was estimated using Kaplan-Meier method. Subgroup analysis was carried out according to metastatic disease and treatment; differences were assessed with the log-rank test. ESMO-GROW (European Society for Medical Oncology Guidance for Reporting Oncology real-World evidence) criteria were applied [3]. All analyses were conducted using SPSS version 22 statistical software (IBM Corp, Armonk, NY).

Ethical considerations

In accordance with Resolution 8430 of 1993, which establishes the scientific, technical, and administrative standards for health research in Colombia, this study is classified within the category of research without risk. Informed consent was not required since the study was a review of medical records, and patients were not subjected to any study-based intervention. Confidentiality was guaranteed by using data masking during the analysis process.

The study was approved by the Comité de Ética para la Investigación Clínica (CEIC) of the Fundación Centro de Investigación Clínica (CIC) on October 26, 2023, as documented in Act 467.

## Results

Baseline characteristics

**Figure 1:**
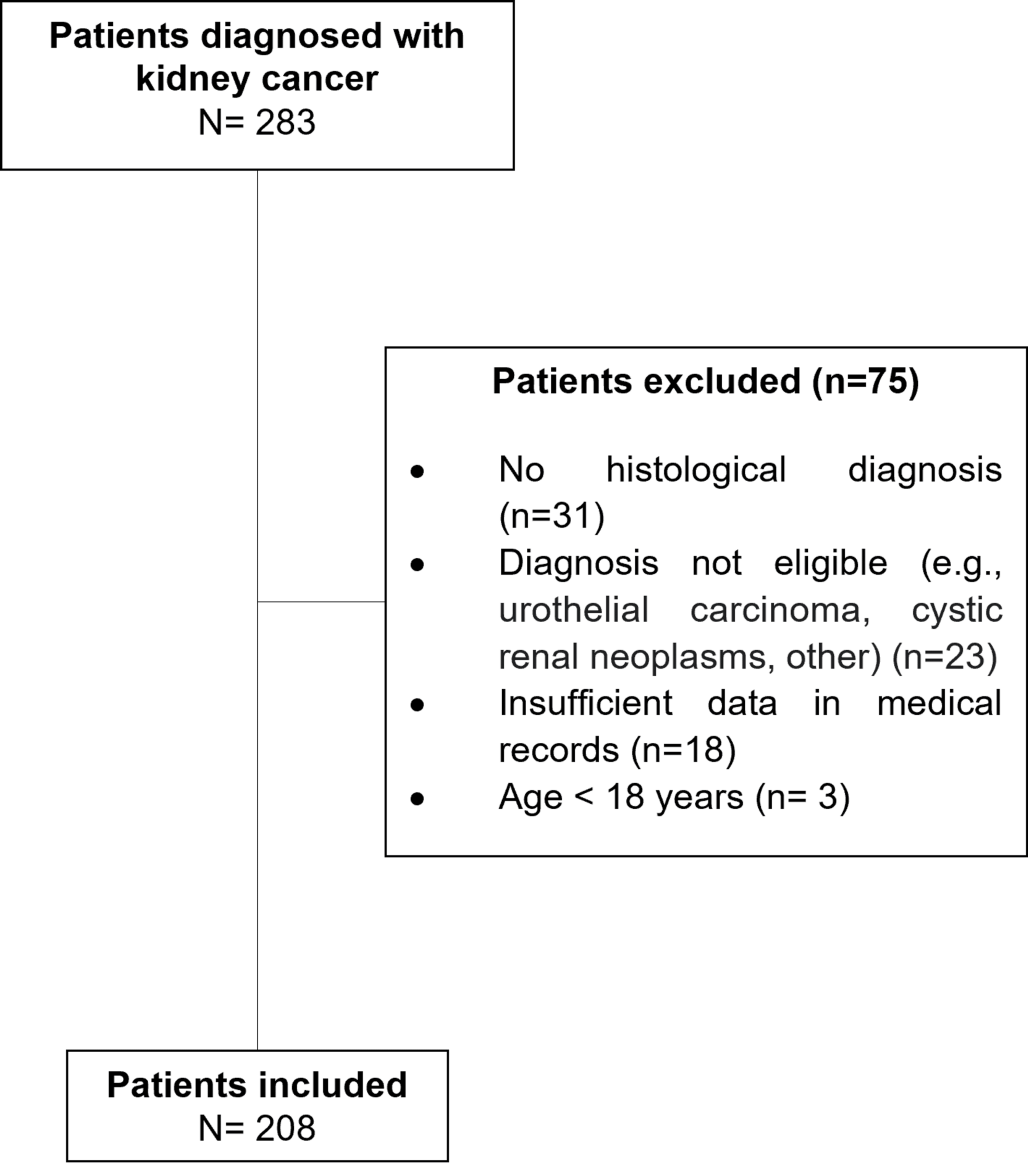
Patient selection for renal cell carcinoma study in Medellín, Colombia.

From 2008 to 2024, 283 patients with RCC were treated at the institution. Of these, 208 patients with RCC met the inclusion criteria (Figure 1).

In our cohort, 114 (54.8%) patients were men, and the median age was 60.4 years (IQR 51.9-69.8 years) at diagnosis. Clear cell renal cell carcinoma (ccRCC) was the histological diagnosis in 167 (80.3%) cases. Stage I and stage IV were assigned to 58 (27.9%) and 48 (23.1%) of cases, respectively. A second primary malignancy was found in 38 (18.3%) patients. Of these, the most common was breast cancer in seven (18.4%) patients (Table 1).

**Table 1:** Sociodemographic and clinical characteristics of patients with renal cell carcinoma. Me: Median. IQR: interquartile range. *The contributive regime is a government-regulated health insurance for formal workers and their families, it is co-financed by employees and employers. The subsidized regime is mainly financed through taxes; there is no contributions from its members. The special regime includes unionized workers of state companies, the police, and the military forces. Private insurance: individuals who can also pay for complementary Insurances or pay directly for healthcare services [4]. †Clear cell and papillary features, collecting ducts, papillary with sarcomatoid differentiation, papillary urothelial carcinoma, clear cell tubulopapillary. ‡For risk classification in metastatic disease, 46 synchronous patients and 61 metachronous patients were included. §Adrenal tumor, GIST (gastrointestinal stromal tumor), Hodgkin lymphoma, Mediastinal tumor, skin tumor.

Characteristics	n (N=208)	%
Age (median (IQR))	60.4 years (51.9-69.8)	
Sex		
Women	94	45.2
Men	114	54.8
Affiliation type to the healthcare system*		
Contributive regime	132	63.5
Subsidized regime	5	2.4
Special regime	4	1.9
Private insurance	67	32.2
Histology		
Clear cell	167	80.3
Clear cells with sarcomatoid features	9	4.3
Papillary	12	5.8
Sarcomatoid	1	0.5
Other†	13	6.3
Non-classified	6	2.9
Stage at diagnosis		
I	58	27.9
II	34	16.3
III	38	18.3
IV (T4)	2	1
Unknown	30	14.4
Risk classification in metastastic disease	(n=107)	51.4
Low risk	43	40.2
Non-low risk	64	59.8
Second primary malignancy		
Yes	38	18.3
Breast cancer	7	3.4
Prostate cancer	6	2.9
Colorectal cancer	5	2.4
Thyroid cancer	4	1.9
Lung cancer	3	1.4
Stomach cancer	2	1
Contralateral kidney cancer	2	1
Bladder cancer	2	1
Other§	7	3.4
No	170	81.7

Treatment

Among patients with early-stage or locally advanced disease (stages I to IV - T4), 126 (95.4%) were treated with nephrectomy followed by surveillance. Nephrectomy followed by surveillance was the treatment for all 30 patients with unrecorded stage. Tyrosine kinase inhibitor (TKI) was the adjuvant therapy in five (100%) cases (Table 2). No patient underwent adjuvant pembrolizumab therapy because it was not approved in Colombia at that time.

**Table 2:** Initial treatment of patients according to stage at renal cell carcinoma diagnosis. TKI: Tyrosine kinase inhibitor; IO: immunotherapy; BVCZ: Bevacizumab; mTOR: mammalian target of rapamycin. *In the group of patients with early-stage disease, five underwent radical nephrectomy, and in one patient, the type of nephrectomy performed is unknown. †In the group of patients with metastatic disease, 20 underwent radical nephrectomy, and in six patients, the type of nephrectomy is unknown. ‡Palliative care without any other anticancer therapies.

Treatment modality	Early-stage / locally advanced disease		Metastatic disease		Unknown stage at diagnosis		Complete cohort	
	n	%	n	%	n	%	n	%
	(n=132)		(n=46)		(n=30)		(n=208)	
Nephrectomy followed by surveillance	126	95.4	3	6.5	30	100	159	76.4
Partial	17	13.5	0	0.0	0	0.0	17	10.7
Radical	86	68.3	2	66.7	16	53.3	104	65.4
Unknown	23	18.3	1	33.3	14	46.7	38	23.9
Nephrectomy followed by systemic therapy	5*	3.8	26^†^	56.5	0	0	31	14.9
TKI	5	100	18	69.2	0	0	23	74.2
IO	0	0	3	11.5	0	0	3	9.7
IO with TKI	0	0	1	3.8	0	0	1	3.2
Interferon with BVCZ	0	0	2	7.7	0	0	2	6.5
Chemotherapy	0	0	2	7.7	0	0	2	6.5
Systemic therapy alone	1	0.8	15	33.3	0	0	16	7.7
TKI	1	100	8	53.3	0	0	9	56.3
IO	0	0	3	20	0	0	3	18.8
IO with TKI	0	0	1	6.7	0	0	1	6.3
Interferon with BVCZ	0	0	1	6.7	0	0	1	6.3
mTOR inhibitor	0	0	2	13.3	0	0	2	12.5
Supportive care^‡^	0	0	2	4.4	0	0	2	1

**Figure 2:**
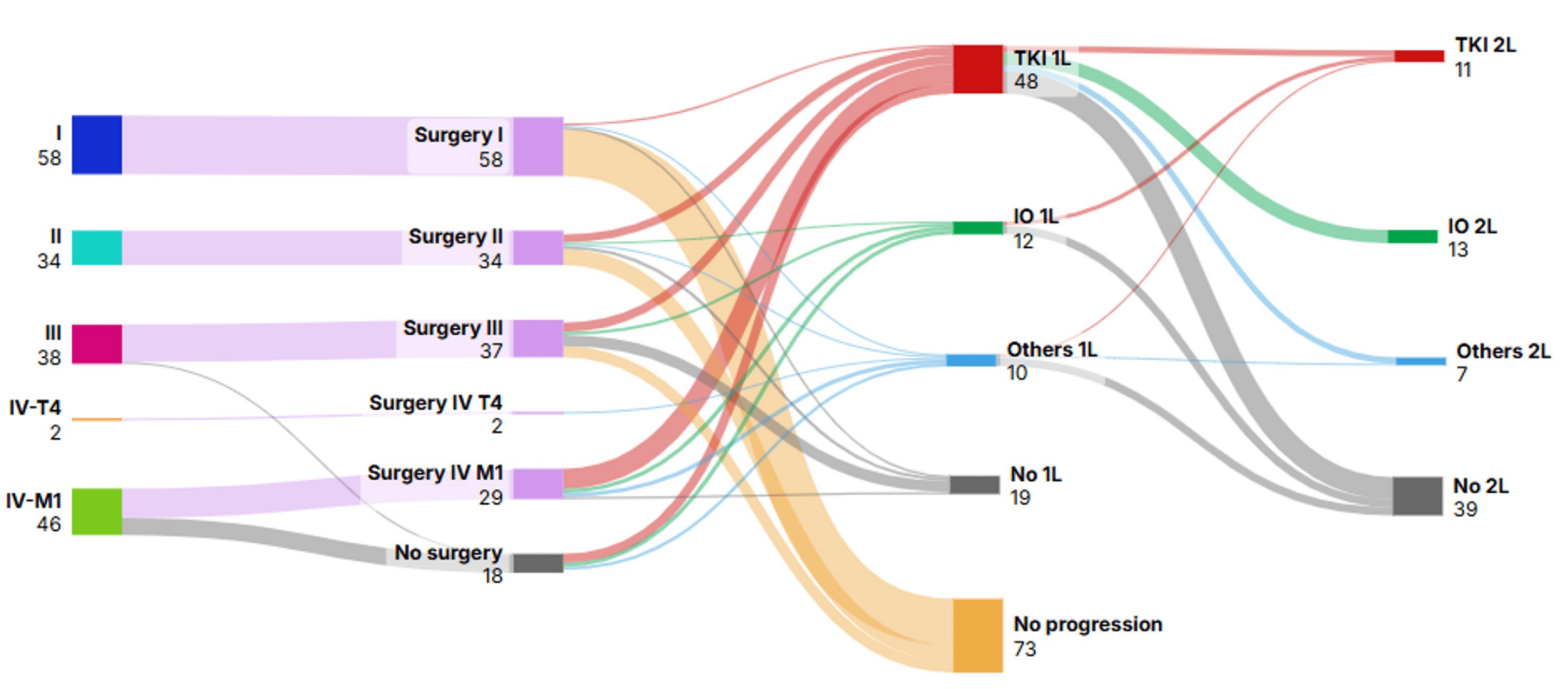
Treatment sequences in patients with renal cell carcinoma by stage. TKI: Tyrosine kinase inhibitor. IO: Immunotherapy. 1L: First line. 2L: Second line.  Others: included chemotherapy, interferon plus bevacizumab, mTOR inhibitors, and interleukin-2.

Patients’ distribution according to initial stage and their pathway through different scenarios of disease are represented in Figure 2.

Treatment for metastatic disease

In the total cohort, 46 patients presented with synchronous disease, while 61 developed metastases following the initial diagnosis (metachronous) (Table 1). Among the synchronous group, 29 (63.0%) patients underwent surgery with curative intent, all of whom subsequently experienced recurrence (Table 2).

Of the 107 patients with metastatic disease, 82 received first-line therapy. Among them, 58 (70.7%) patients were treated with TKI, 12 (14.6%) with immunotherapy (IO), and 12 (14.6%) with other systemic treatments, including chemotherapy, mTOR inhibitors, or interferon. A second line of treatment was administered to 34 patients (31.8%). In this scenario, 14 (41.2%) patients received TKI, 14 (41.2%) IO, four (11.7%) mTOR inhibitors, and two (5.9%) interferon.

**Figure 3:**
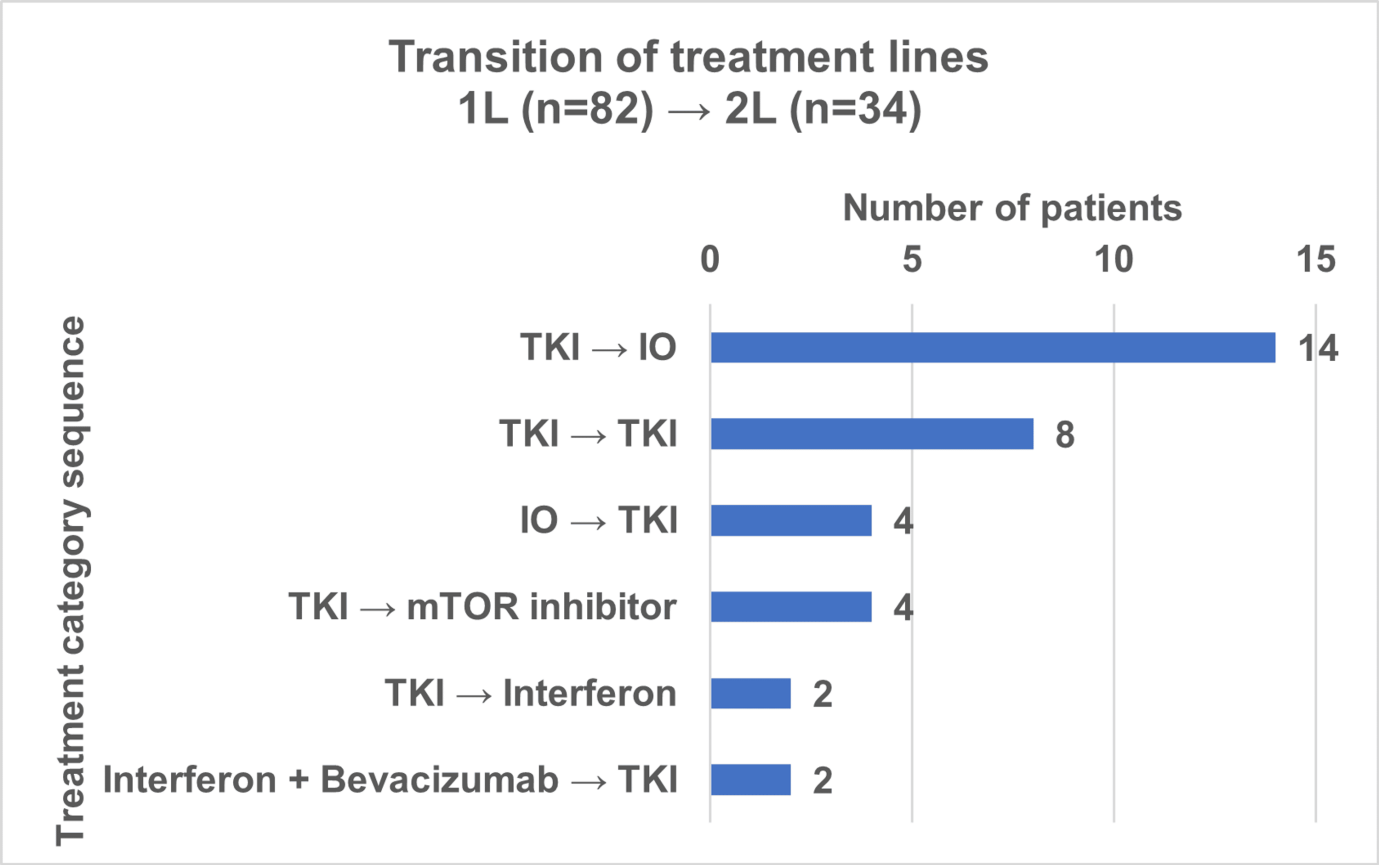
Transition from first- to second-line therapy in metastatic renal renal cell carcinoma. TKI: Tyrosine kinase inhibitor; IO: immunotherapy; mTOR: mammalian target of rapamycin.

Among the 34 patients who received a second-line regimen, the most common sequences were TKI followed by IO (n=14, 41.2%) and TKI followed by TKI (n=8, 23.5%). The aforementioned factors were responsible for approximately two-thirds of all treatment transitions (Figure 3).

Outcomes

**Figure 4:**
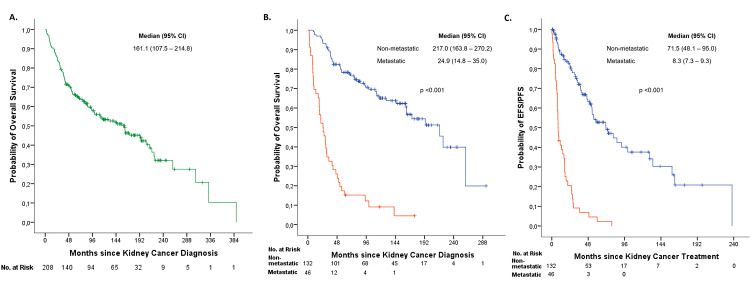
Kaplan–Meier curves for: (A) overall survival of the entire patient cohort, (B) overall survival by subgroups according to initial stage (non-metastatic vs. metastatic), (C) event-free survival (EFS) and progression-free survival (PFS) in patients with renal cell carcinoma..

The median follow-up was 84.6 months (IQR 37.7-161.8) from diagnosis. At the time of analysis, 97 patients (46.6%) were alive. Median OS for the entire cohort was 161.1 months (95% confidence interval (CI): 107.5-214.8) (Figure 4A). Based on the stage at diagnosis, median OS was 217.0 months (95% CI: 163.6-270.4) for patients with early-stage or locally advanced disease (classified as non-metastatic). In contrast, patients presenting with metastases at diagnosis had a median OS of 24.9 months (95% CI: 14.3-35.5) (Figure 4B). A significant difference in OS was found between stage groups (p <0.001).

Among the 132 patients in the non-metastatic group, 59 (44.7%) experienced disease progression, whereas 45 (97.8%) of the 46 patients in the synchronous group experienced progression. Median EFS and PFS were 71.5 months (95%CI: 45.2-97.9) and 8.3 months (95%CI: 6.7-9.9), respectively (Figure 4C). A significant difference in EFS/PFS was observed between groups (p<0.001).

**Figure 5:**
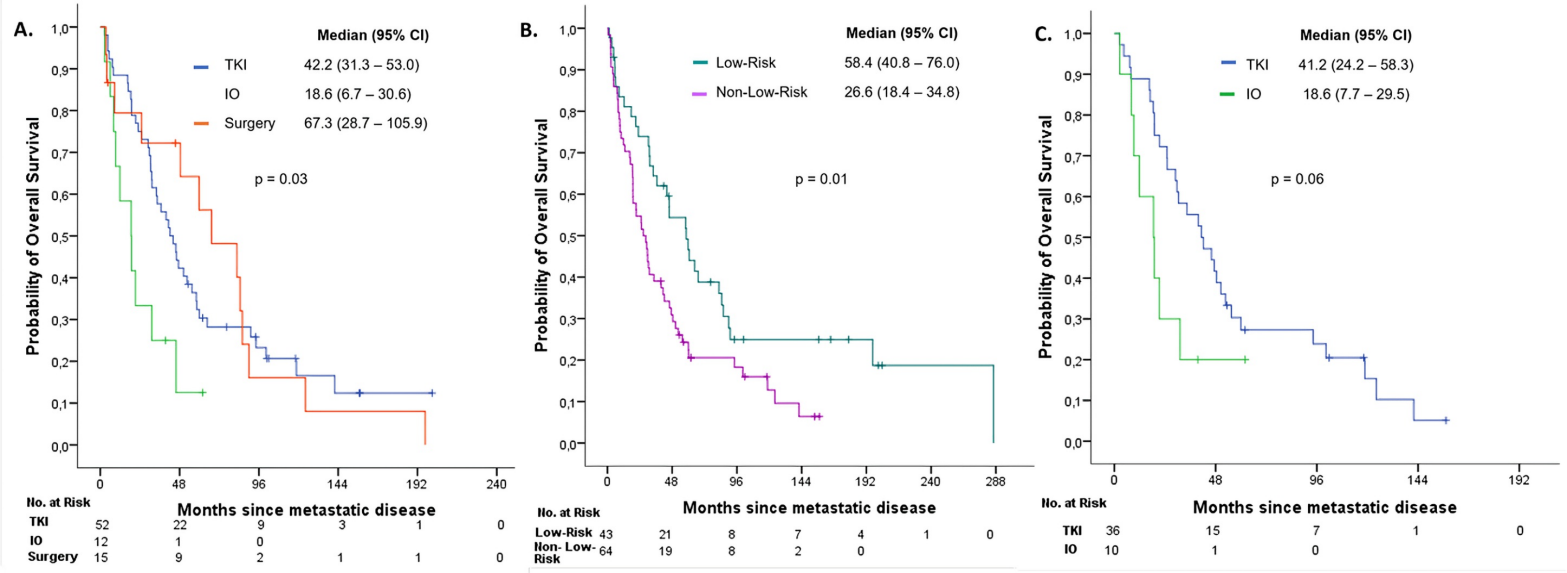
Kaplan–Meier curves for: (A) overall survival in patients according to the type of treatment received in metastatic disease, (B) overall survival by risk category in metastatic disease, and (C) overall survival within the non-low-risk group in metastatic disease by treatment (TKI vs. IO).

Overall survival was assessed in patients who developed metastases (either synchronous or metachronous) and was stratified by first-line treatment for metastatic disease: TKI, IO, or surgery. Median OS was 42.2 months (95% CI: 31.3-53.0), 18.6 months (95% CI: 6.7-30.6), and 67.3 months (95% CI: 28.9-105.9), respectively. The differences observed among the groups were found to be statistically significant (p=0.03) (Figure 5A).

When further stratified by risk category, median OS was 58.4 months (95% CI: 40.8-76.0) in the low-risk group versus 26.6 months (95% CI: 18.4-34.8) in the non-low-risk group (p=0.01) (Figure 5B). Within the non-low-risk group, median OS was 41.2 months (95% CI: 24.2-58.3) for TKI and 18.6 months (95% CI: 7.7-29.5) for IO (p=0.06) (Figure 5C).

## Discussion

This study provides one of the largest real-world descriptions of RCC in Colombia, providing insight into the demographic, clinical, and survival characteristics of patients treated at a specialized outpatient oncology center.

The median age at diagnosis in our cohort (60.4 years) was lower than the global median of 75 years reported in international registries [5,6]. This finding may reflect regional demographic patterns, potential differences in risk factor exposure, or earlier access to specialized care in a proportion of patients. In accordance with international data, the majority of cases were identified as clear cell RCC, which remains the most prevalent histological subtype worldwide [7].

In our cohort, the male-to-female distribution was nearly balanced (~55% men and ~46% women), which contrasts with the global epidemiological trend, which has been shown to exhibit a clear male predominance, with reported ratios of approximately 2:1 [8,9].

The proportion of patients diagnosed with advanced or metastatic disease in our cohort (22.1%) was comparable to figures reported in the literature, ranging from 17% in some series to approximately 25%-30% in others [8,10].

Treatment patterns in this study reflect the evolution of therapeutic availability in Colombia over the past decade. Nearly all patients with localized disease underwent nephrectomy, consistent with international standards of care [11,12]. In contrast, systemic therapy for metastatic disease was dominated by TKIs, with relatively limited use of immunotherapy. This pattern differs from current global practice, where immune checkpoint inhibitors (ICI), either alone or in combination with TKIs, are standard first-line therapy for advanced disease [13-15]. Many of the patients included in this analysis were treated in the TKI era prior to the ICI era. Additionally, delays in regulatory approvals in Colombia restricted access to ICIs. For example, in Colombia, there is still no regulatory approval of adjuvant pembrolizumab for resected high-risk RCC.

In our data, 46.3% of the patients did not receive a second-line agent for advanced RCC. The limited access to second-line therapy in our study is similar to that reported in two cohorts of patients with advanced kidney cancer in the United States and Rumania, where second-line treatment was reported in 48.4% and 51.3% of cases, respectively [16,17]. Given that a substantial proportion of patients may not have the opportunity to receive second-line therapy, the careful selection of the initial treatment strategy acquires even greater clinical relevance. It is imperative to underscore the recommendation of administering very active RCC treatments as a primary course of action, given that approximately half of the patients will only receive a first-line therapy.

Survival outcomes in our cohort are generally consistent with those reported in international series, although median OS in non-metastatic patients was notably long. This phenomenon may be partially attributed to several factors, including a relatively younger population, the high rate of surgical management, and the long follow-up period achieved in this study. In the metastatic subgroup, the median PFS of 8.3 months with first-line therapy is comparable to outcomes reported in the pre-immunotherapy era with TKIs alone [18,19].

A statistically significant discrepancy was identified in survival outcomes between patients with low-risk and non-low-risk disease. The median overall survival was 58.4 months versus 26.6 months, respectively. Among patients in the non-low-risk group, those treated with TKIs exhibited a numerically longer median survival of 41 months compared with 18.6 months in those treated with immunotherapy, although this difference did not reach statistical significance. Both results may be attributable to the small sample size within immunotherapy group.

An additional relevant observation was the occurrence of second primary malignancies in nearly one-fifth of patients, with breast cancer being the most frequent. While this phenomenon has been described in other series [20], its clinical implications remain uncertain. It raises questions regarding potential shared risk factors, genetic predispositions, or treatment-related effects that warrant further exploration in larger, multicenter studies.

This study has several limitations inherent to its retrospective and single-center design. Missing data in some variables, particularly stage and treatment details, could have introduced bias. Furthermore, therapeutic options have changed substantially over the long study period, limiting the direct comparability of survival outcomes with current international standards. Nonetheless, the strengths of this work include the relatively large sample size, accurate characterization of the patient population in terms of demographic variables and TNM stage, long follow-up, and robust survival data verified through national registries.

Another limitation of the study is that, due to the retrospective nature of the data, information on prognostic risk status according to the International Metastatic Renal Cell Carcinoma Database Consortium (IMDC) was not consistently available in the medical records. This prognostic classification is essential to better understand and accurately interpret treatment selection strategies in metastatic disease. We tried to minimize the impact of this shortcoming by creating the low- and non-low-risk categories for metastatic and recurrent disease to provide a rough estimate of risk distribution.

In Colombia, epidemiological statistics can be obtained from government sources and population-based registries (incidence, prevalence, and mortality) for patients with RCC [21,22]. However, to the best of our knowledge, there is an absence of other comprehensive description of RCC in Colombia, and it can be a reference point for future studies as a baseline. Navarro et al. published a description of 40 consecutive stage I-III RCC patients treated in Barranquilla, Colombia [23]. In this study, five-year follow-up was available in only 15 patients, and there were no stage IV patients included. In contrast, our dataset has a median follow-up of 85.6 months, which can accurately depict the long-term survival patterns of the patient population. It also included 45 stage IV RCC patients, adding valuable information on the outcome of this critically important subgroup for the clinical oncologic community.

## Conclusions

In conclusion, this study found that demographic and clinical features of RCC patients in Colombia mirror global patterns. Therapeutic limitations, particularly restricted access to modern immunotherapy, continue to represent a major gap in the management of metastatic disease. Future efforts should focus on expanding access to contemporary systemic therapies, exploring strategies for earlier detection, and generating multicenter data to better characterize outcomes in the region.

This institution-based real-world study of patients with RCC diagnosis, treated at a single outpatient oncology center in Colombia, provides a comprehensive characterization of demographics, data, stage distribution, treatment patterns, and survival outcomes.
